# Variables influencing executive functioning in preschool hearing-impaired children implanted within 24 months of age: an observational cohort study

**DOI:** 10.1007/s00405-020-06343-7

**Published:** 2020-09-11

**Authors:** Maria Nicastri, Ilaria Giallini, Martina Amicucci, Laura Mariani, Marco de Vincentiis, Antonio Greco, Letizia Guerzoni, Domenico Cuda, Giovanni Ruoppolo, Patrizia Mancini

**Affiliations:** 1 Department of Sensorial Organs, Sapienza University of Rome, Viale dell’Università, 31, 00161 Rome, Italy; 2 Department of Oral and Maxillofacial Sciences, Sapienza University of Rome, Rome, Italy; 3 Department of Otorhinolaryngology, “Guglielmo da Saliceto” Hospital, Piacenza, Italy

**Keywords:** Cochlear implants, Child, Preschool, Executive functions, Working memory

## Abstract

**Purpose:**

Executive Functions (EFs) are fundamental to every aspect of life. The present study was implemented to evaluate factors influencing their development in a group of preschools orally educated profoundly deaf children of hearing parents, who received CI within 2 years of age.

**Methods:**

Twenty-five preschool CI children were tested using the Battery for Assessment of Executive Functions (BAFE) to assess their flexibility, inhibition, and non-verbal visuo-spatial working memory skills. The percentage of children performing in normal range was reported for each of the EF subtests. Mann–Whitney and Kruskal–Wallis were performed to assess differences between gender, listening mode, and degree of parents’ education subgroups. The Spearman Rank Correlation Coefficient was calculated to investigate the relationship between EF scores of audiological and linguistic variables.

**Results:**

Percentages ranging from 76 to 92% of the children reached adequate EF scores at BAFE. Significant relations (*p* < 0.05) were found between EFs and early intervention, listening, and linguistic skills. Furthermore, CI children from families with higher education level performed better at the response shifting, inhibitory control, and attention flexibility tasks. Economic income correlated significantly with flexibility and inhibitory skills. Females performed better than males only in the attention flexibility task.

**Conclusions:**

The present study is one of the first to focus attention on the development of EFs in preschool CI children, providing an initial understanding of the characteristics of EFs at the age when these skills emerge. Clinical practice must pay increasing attention to these aspects which are becoming the new emerging challenge of rehabilitation programs.

## Introduction

Executive functions (EFs) are a set of top-down cognitive skills, which include response inhibition, self-control, interference control, working memory, and cognitive flexibility. These mental processes provide critical support for learning and development, allowing us to retain and process information in our brain, focus our attention, filter distractions, and switch mental gears. Longitudinal studies have highlighted that EFs are at the core of school performance, emotional regulation, and social, moral, and communication skills [1, 2].

Language plays a fundamental role in EF development as it allows children to share elements critical for the elaboration and expansion of mental images, facilitates the adaptation to environment requests, and guarantees the inhibition of impulsive acting [3]. Good language knowledge is necessary to develop working memory, the executive function at the base of many cognitive operations, as it allows children to code external information to be then processed, stored, maintained, retrieved, and transformed into phonological and lexical representations for use in a range of different processing tasks [4]. Furthermore, the internal use of language, through self-reflection and self-questioning supports sustained and shifted attention, formation of rules and plans, and control of behavior during problem-solving activities [5].

Given the relationship between language and EFs, deaf and hard‐of‐hearing (DHH) children represent an at-risk category for the development of these skills. Their limitations in receiving auditory information, accessing spoken language, and using language for communicative purposes could affect their participation in daily communicative interactions from birth, negatively influencing the neural organization and the development of domain-general neurocognitive skills that rely on auditory experiences, speech perception, and spoken language processing [6].

As a matter of fact, in the past 20 years, most of the studies carried out into school age deaf population have highlighted a general delay in several areas of executive functioning, such us verbal working memory [7–12], visual sequence learning [13], verbal rehearsal and fluency speed [7, 8, 11, 14], emotional and impulse regulation and inhibition–concentration skills [7–11, 15–19], sustained attention and attention shifting [11, 15, 19], sequential processing [9, 12], problem-solving and planning abilities [9, 15, 16, 19].

Poor executive skills were detected both in DHH children using hearing aids [15, 16] and those with cochlear implants [7–14, 18].

In preschool children with CI, EF skills were only studied by Beer et al. [10]. A sample of 24 DHH children who were implanted prior to age 3 and a control group of NH peers were directly assessed for short-term working memory, inhibition, and organization-integration skills. Furthermore, the Behavior Rating Inventory of Executive Function-BRIEF for parents (preschool version) was used to measure EF behaviors in everyday life [20].

Children’s assessments showed significant differences between CI and NH in the domain of inhibition–concentration measures only, with a quarter of CI falling in the clinical range, against zero of NH peers.

For BRIEF, parents reported inhibitory control and working memory problems in almost 50% CI against the 15–30% of NH children. However, no statistically significant differences were reported in organization/planning. At the bivariate analysis, among all demographic and audiological factors, the duration of CI use was the only factor to correlate with fewer problems for planning and organization based on the parent-reported Plan/Organize scores of BRIEF. Language skills were positively associated with working memory and planning/organization skills, as measured through the BRIEF.

The preschool age represents the moment when core EFs are establishing, and the skills mastered in this period are strongly related to attentiveness, concentration, self-control, and ability to cope with stress and frustration during late childhood and adolescence [21] as well as physical health, financial well-being, and criminal outcomes in adulthood [21]. Investigating such an early phase of competence development could be useful in developing adequate strategies of intervention aiming to reduce the long-term negative effect of early inadequate patterns of EF skills. Given the paucity of data on this phase of development, the present study was implemented to evaluate factors influencing the development of adequate EFs in a group of preschool orally educated profoundly deaf children of hearing parents, who received CI within 2 years of age.

## Materials and methods

### Participants

Children with congenital profound deafness (Pure-Tone Average in the better ear ≥ 90 dB HL for 500–4000 Hz), aged 3–6 years at the time of enrollment, were included. To limit the number of variables that can be reasonably accounted for in the statistical analysis without introducing confounding interactions and to eliminate variation in the confounders [22], the following inclusion criteria were introduced in subject selection: normal cognitive level, as assessed by Raven Colored Progressive Matrices [23]; absence of additional handicap and/or associated disorders verified by clinical history and neuropsychiatric evaluation; absence of pathologies/alterations that could impact the auditory outcomes of cochlear implant, such as cochlear and nerve malformations, auditory neuropathy, meningitis; Italian as primary household language; child oral education setting.

Economic income was defined on the basis of the Italian economic family status indicator index named ISEE (Indicatore della Situazione Economica Equivalente: Equivalent Economic Situation Index). The ISEE index allocates economic income brackets based on annual income, real estate assets, number of members of the family, and city of residence (https://www.inps.it/nuovoportaleinps/). Based on this index, three economic income brackets were defined: low, middle, and high. Parents education was classified in years of schooling [middle (8), high (13) and university degree (18)].

### Study design

The present study was structured as a cohort longitudinal study and data were collected in two CI centers: Policlinico Umberto I Hospital, Rome and “Guglielmo da Saliceto” Hospital, Piacenza, Italy. The protocol was approved by the local ethics committees of the two hospitals. The recruited families gave written informed consent for the assessment of their child before commencing any study-related procedure. Protocol studies were approved by Institutional Review Board and were conducted according to the principles and rules laid down in the Declaration of Helsinki and its subsequent amendments.

### Assessment

#### Auditory skills

Speech recognition was assessed using standard Italian phonetically balanced bisyllabic words for paediatric populations [24]. A 10-item list was preceded by a practice list. Items were administered in a soundproof room, via a loudspeaker placed at 1 m distance from a table where the child was sitting next to a speech therapist. Stimuli were presented in quiet at 65 dB SPL 0° azimuth. Score was calculated as a percentage of words correctly repeated.

General auditory receptive abilities were assessed with Categories of Auditory Performance-2 (CAP-2), developed to rate outcomes for paediatric cochlear implants in everyday life [25]. The index is a reliable measure of perceptive outcome in developing children, with a good inter-user reliability (correlation coefficient > 0.75), and provides a scale on which auditory abilities can be rated in nine categories in order of increasing difficulty, ranging from no awareness of environmental sounds (category 0) to use of phone with unknown speaker in unpredictable context (category 9).

#### Language skills

Children were tested individually in a quiet room, by two trained speech therapists. Tests were conducted using spoken language as all the children communicated orally. Two Italian Standardized Language tests were used to assess, respectively, lexical comprehension and lexical production and morpho-syntactic comprehension.

Lexical comprehension and production were measured using specific subtests of “Test di Valutazione del Linguaggio -TVL” [Test for Evaluation of Language] for preschooler [26] designed for children ranging from 3 to 6 years of age. Children are presented real objects or pictures and were requested to point or name them. The raw score is represented by the number of correct responses, which are then transformed into normative weighted scores ranging from 0 to 10. A weighted score of < 3 is considered below the average (more than − 1 sd). Test–retest reliability was, respectively, 0.91 and 0.96. for comprehension and production.

Morphosyntactic comprehension was assessed using “Prove di Valutazione della Comprensione Linguistica-PVCL” [Test for the Evaluation of Linguistic Comprehension] [27]. PVCL is a test that investigates grammatical comprehension skills (for example reflexive, negative, passive, reversible, temporal, causal, conditional, and adversative phrases). It is divided into protocols ordered by age groups (from 3.5 to 7 years). Each test stimulus is presented in a four-picture, multiple-choice format with lexical and grammatical distractors. For each item, the examiner reads a sentence that refers to one of four drawings. Children are asked to point to the drawing corresponding to the sentence presented by the examiner. A total raw score is calculated based on the number of correct items identified, each of which has a different value according to its developmental complexity. The raw score is then converted into a percentile. A percentile < 25 is considered below average (more than -1sd). Test–retest reliability was 0.93.

#### Executive function skill assessment

The neuropsychological assessment of EFs was carried out using the Battery for Assessment of Executive Functions-BAFE [28], a battery specifically designed to assess nuclear EFs in preschool children, aged from 3 to 6. It is composed of four subtests, consisting of quick, easy, and quite engaging activities for young children, with the aim of measuring three aspects of EF skills: Flexibility, Working Memory, and Inhibition.

Flexibility (set-shifting) refers to the ability to shift, in a flexible manner, between different mental plans to reflect different situations or external requests. Usually, subjects with reduced set-shifting when faced with a problem or new situation, show perseverant behaviors, mental rigidity, and lack or reduction of flexibility. Flexibility is thought to be subdivided into two different skills: response shifting flexibility and attention flexibility. Response shifting flexibility is the ability to shift behavior from one mental set to another one that conflicts with the first (i.e., resolution of cognitive conflicts). Attention flexibility is the ability to focus attention on a mental set while resisting interference. BAFE assesses these two aspects of flexibility, including two different subtests: card sort, for assessing response shifting flexibility, and triplets of circles pattern making, for assessing attention flexibility. Both subtests evaluate flexibility with minimum language skills involvement.

For card sort tasks, the child is shown images that they have to categorize at first according to shape and then according to color. The images must be inserted in the appropriate containers. One point is given for each complete and correct answer (range 0–3): a complete answer is when the two criteria, shape and color, are present in the sample image (little blue bear and red house → little red bear and blue house → 1 point). The set-shifting ability is examined through the child’s ability to switch from one categorization criterion to another during the test. One point is given for each correct response (range 0–3).

For triplets of circles pattern making, the child is shown a series of colored circles printed on a strip of card, which are always repeated with the same sequence, and they are asked to name the color of each circle (for example “blue, blue, red, blue, blue, red”) for all of the sequences. The child is then requested to reproduce the overall sequence on a board using small plastic blue and red circles. One point is given for each correct triplet (range 0–6).

Inhibition refers to the ability to stop or delay impulsive/compelling responses, to self-control attention and emotions to achieve a behavioral adaptation goal. Moreover, it encompasses delay aversion or gratification skills and, more generally, the ability to wait. Usually, children with low inhibitory control show impulsivity and inefficient organization.

In BAFE, inhibitory skills are assessed by subtest Stroop-like day-night. It consists of a set of cards, presented to the child, where the moon or sun is alternatively depicted. The child is asked to say the word “day” when he is shown images with the moon, and the word “night” when he is shown ones with the sun. The child must inhibit automatic responses. One point is given for each correct answer (range 0–16).

Working memory refers to the ability to store and manage verbal and/or non-verbal information, to reflect complex cognitive tasks such as understanding, learning, and reasoning. A deficit in working memory makes it harder to remember information, to plan actions to achieve a goal, to create mental representations, and to make decisions. Working memory has a crucial role in the selection, initiation, and termination of information-processing functions such as encoding, storing, and retrieving data. In BAFE, working memory is assessed in its visuo-spatial subdomain by subtest Spin the Pots.

The test is administered using a rotating tray with eight different colored cups. A red token is placed under each cup and a cloth is used to cover the game. The tray is rotated, while it is covered. After it is rotated, the child is asked to lift the cloth and choose a cup to find a red token. The child has to recover all of the tokens, but without choosing the same cup more than once. The position of the cups changes each time, because, after a red token is recovered, the cups are rotated on the supporting tray. The raw score is given by the number of attempts made to recover all tokens (range 8–16).

Raw scores for each FE subtest are converted into percentile ranking according to the normative sample of the test. The BAFE manual references normal scores as ≥ 25 percentile.

The battery is standardized on a sample of 358 NH children (167 girls, 191 boys), balanced for gender and subdivided into six age groups (53 children in the 36–42 month group; 51 children in the 43–48 month group; 61 children in the 49–54 month group; 60 children in the 55–60 month group; 89 children in the 61–66 month group, and 44 children in the 67–72 month group). BAFE shows a good reliability coefficient for subtest Card Sort and Spin the Pots (KR-20 = 0.77), and excellent reliability coefficients for subtest Day and Night (KR-20 = 0.92) and subtest Triplets of circles (KR-20 = 0.94).

### Statistical analysis

Statistical descriptive analysis is presented as median [min. and max.] for continuous variables. CI outcomes were compared with scale norms for the test batteries (which are based on nationally representative samples for typically developing, normal-hearing children). The percentage of children performing in normal range was reported for each of the EF subtests. Consistently with the non-normal distribution of data, the Mann–Whitney and Kruskal–Wallis tests were used to assess differences between gender, listening mode (mono and bilateral users), and degree of parents education. The Spearman Rank Correlation Coefficient was calculated to investigate the relationship between EF scores and demographic, audiological, and linguistic variables. *P* values less than 0.05 in either direction were considered as significant. Statistical analysis was carried out using a PC version of Statistical Package for Social Sciences 25.0 (SPSS, Chicago, IL, USA).

## Results

### Subjects

Twenty-five children (15 females and 10 males) met the inclusion criteria. They had a median age at the assessment of 5 years (min 3.3–max 5.9 years). Table 1 shows the main demographic and clinical characteristics.

**Table 1:** Demographic and clinical characteristics of the study population (*n* = 25), expressed as number (%) for categorical and as median (min–max) for continuous variables

Categorical variables			*n* (%)
Gender		Female	15 (60%)
		Male	10 (40%)
Listening mode	Monoaural CI		8 (32%)
	Bilateral CI	Simultaneous	7 (28%)
		Sequential	10 (40%)
Cochlear implants	Cochlear^®^		14 (56%)
	AB^®^		11 (44%)
Strategies	ACE™		14 (56%)
	Hi-Res120™		11 (44%)

Continuous variables		Median	[Interval]
Age at assessment (years)		5	(3.3–5.9)
Age at diagnosis (months)		4	(1–21)
Age at cochlear implant (months)		12	(8–24)
Hearing Age (months)		48	(15–59)
CPM normal score		82	(42–100)
Bisyllabic word recognition (%)		100	(70–100)
CAP-2		7	(5–7)
Lexical comprehension (weighted score)		6	(1–8)
Lexical production (weighted score)		5	(1–8)
Morpho-syntactic comprehension (percentile)		75	(5–80)
Mother’s level of education (years)		13	(8–18)
Father’s level of education (years)		13	(8–18)

All children had a profound congenital sensorineural hearing loss caused by Connexin 26 mutation (11), ototoxicity (4), and unknown etiology (11). Median chronological age at diagnosis was 4 months (range 1–21). All children started a habilitation process within 1 month of diagnosis. Median age at implantation was 12 months (range 8–24). Sixty-four percent of children were diagnosed within 6 months and implanted within 12 months of age. Of them, 50% received CI between 8 and 12 months of age. The median hearing age at assessment was 48 months (range 15–59). Fourteen recipients were implanted with Cochlear devices programmed with ACE strategy and 11 with Advanced Bionics devices programmed with Hi-Resolution 120 strategy. Seventeen children used bilateral CIs (7 simultaneous and 10 sequential), while 8 were unilateral CI users. Daily use of the devices was assessed through parental reports, as data logging was not available for most children using speech processors without this feature (e.g., AB Neptune speech processor). All children have been reported to use their device for more than 10 h a day.

The median normalized score for non-verbal intelligence at CPM was 82 percentile (range 42–100). Concerning communication mode, all children attended oral rehabilitation programs and were completely immersed in an oral communication environment. All of them used oral spoken language and attended normal mainstream kindergarten with the presence of a support teacher, according to the normal legislative procedure of the Italian Ministry of Education.

Median bisyllabic word recognition in quiet was 100 (range 70–100) and the CAP-2 category score ranged from 5 (understanding of common phrases without lip reading) to 7 (use of telephone with known listener). Eighty-eight percent of children had a maximum score in bisyllabic word recognition and high CAP-2 category.

TVL median weighted scores for lexical comprehension and production were, respectively, 7 (range 1–9) and 5 (range 1–9). Eighty-eight percent of children fell into the normal range for lexical comprehension, and 76% showed normal scores for lexical production. The median percentile score for morpho-syntactic comprehension (Rustioni test) was 75%: Ninety-two percent children fell in the normal range. Concerning parents’ education, the median value for school attendance was 13 years (range 8–18) equivalent to a high school level, and 40% of mothers and 36% of fathers had a university degree. Most of the sample was constituted of families with medium economic income (72%). Between the remaining families, 4 (16%) and 3 (12%) had, respectively, a lower or a higher economic income.

### Executive function domains

The scores of each CI child were compared with those reported in the BAFE manual. Each CI children was compared with the NH children belonging to the same chronological age group and the normative score in percentile was computed as indicated by the manual.

The median score in the card sort task, for response shifting flexibility, was 99 percentile (range 10–99), with 22 CI children (88% of recipients) showing the adequate performance (≥ 25 percentile): of these, 18 (72%) obtained an optimal score (≥ 70 percentile). A similar trend was shown in the triplets of circles task, for attention flexibility: median score was 99 percentile (range 5–99), with 23 CI children (92%) reaching sufficient scores and 22 of these (88%) within optimal range performance.

Concerning the Stroop-like day-night, for inhibitory control, median score was 48 percentile (range 5–99), with 19 CI children (76% of recipients) performing in the range ≥ 25 percentile. Seven children (28%) showed high-level performance.

Finally, median score for the Spin the Pots task for visuo-spatial working memory was 43 percentile (range 5–99), with 20 CI children (80% of recipients) showing a normal range performance. Four children (16%) obtained optimal working memory score.

Children who were identified within 6 months of age and received CI within 12 months of age reached ≥ 25 percentile performance in 94% of cases for response shifting flexibility and inhibitory control and in 100% of cases for attention flexibility and visuo-spatial working memory skills. Children who received diagnosis and were implanted later showed more variable performance, with fully sufficient skills in 78% of cases for response shifting and attention flexibility tasks and only 44% for inhibitory control and visuo-spatial working memory.

### Factors that affect EFs

Bivariate correlation was performed between EF outcomes and children’s demographic (age at diagnosis, age at cochlear implantation, hearing age, bi-monolateral CI, economic income, and years of parents’ schooling), audiological (bisyllabic word recognition in quiet), and linguistic variables (lexical comprehension and production and morpho-syntactic comprehension) (see Tables 2, 3).

**Table 2:** Spearman correlations between EFs and CI data, listening skills, and language skills

	Age at diagnosis		Age at CI		Hearing age		Bisyllabic words		CAP-2		Morphosyntactic comprehension		Lexical comprehension		Lexical production	
	*ρ*	*p*	*ρ*	*p*	*ρ*	*p*	*ρ*	*p*	*ρ*	*p*	*ρ*	*p*	*ρ*	*p*	*ρ*	*p*
Response shifting flexibility	− 0.76	** < 0.001**	− 0.72	** < 0.001**	0.62	**0.001**	0.64	**0.001**	0.88	** < 0.001**	0.76	** < 0.001**	0.63	**0.001**	0.67	** < 0.001**
Attention flexibility	− 0.63	**0.001**	− 0.63	**0.001**	0.4	**0.049**	0.63	**0.001**	0.68	** < 0.001**	0.73	** < 0.001**	0.64	**0.001**	0.67	** < 0.001**
Inhibitory Control	− 0.5	**0.011**	− 0.63	**0.001**	0.38	0.62	0.32	0.11	0.36	0.078	0.58	**0.002**	0.66	** < 0.001**	0.69	** < 0.001**
Visuo-spatial WM	− 0.32	0.12	− 0.34	0.10	0.30	0.14	0.29	0.15	0.52	**0.008**	0.50	**0.01**	0.55	**0.004**	0.49	**0.014**

Statistically significant values were set at *p* < 0.05 and are highlighted in bold

**Table 3:** Mann–Whitney and Kruskal–Wallis tests for detecting differences linked to gender, listening mode (mono vs bilateral CI), parents’ level of education, and economic income

	Gender^a^		Listening mode^a^		Mother schooling^b^		Father schooling^b^		Economic income^b^	
	*U*	*p*	*U*	*p*	*H*	*p*	*H*	*p*	*H*	*p*
Response shifting flexibility	54.5	0.1	65	0.8	12.1	**0.002**	9.3	**0.009**	17.4	** < 0.001**
Attention flexibility	40.5	**0.02**	67	0.9	9.7	**0.008**	6	**0.05**	12.1	**0.002**
Inhibitory control	58.5	0.3	49.5	0.3	6	**0.05**	7.2	**0.03**	8.4	**0.015**
Visuo-spatial WM	71.5	0.8	64	0.8	1.8	0.4	3	0.2	4.1	0.1

Statistically significant values were set at *p* < 0.05 and are highlighted in bold

^a^Mann–Whitney test

^b^Kruskal–Wallis test

Language skills correlated significantly with all the EF outcomes. Age at diagnosis and at CI showed negative correlation with inhibitory control and both response shifting and attention flexibility, while they seemed not to impact visuo-spatial working memory. Children with longer hearing age showed better skills in flexibility tasks only. Listening skills were correlated with EFs, with the exception of visuo-spatial working memory.

The Mann–Whitney test revealed no differences in performance due to gender with the exception of the attention flexibility task which was significantly better for females. No significant differences were found between mono and bilateral CI users for any of the EF tasks. The Kruskal–Wallis test showed that children from families with parents who had had secondary school education and a university degree (respectively, 13 and 18 years of education) performed better at the response shifting flexibility task, inhibitory control, and attention flexibility. Economic income correlated significantly with flexibility and inhibitory skills.

## Discussion

The aim of the present study was to evaluate factors that influence EF skills in preschool congenital profoundly deaf children, who received CI within 2 years of age.

The first variables studied were early diagnosis and early cochlear implantation. The behavioral flexibility, attention flexibility, and inhibition skills significantly correlated with both age at diagnosis and age at implantation: children who were diagnosed and implanted early had better skills and were more likely to perform within normal range. Our findings differ from those described by Beer et al. [10] in preschool children and from other studies on school-aged children [8, 9, 12, 14], while are in agreement with data reported by Conway et al. [13]. Beer et al. [10], Kronenberger et al. [8, 9], Davidson et al. [12], and Pisoni and Cleary [14] included in their analysis children with a different age range of onset of deafness (0–36 months), variable pre-implant PTA (ranging from about 70 to 120 dB), auditory neuropathy, and ear congenital malformations. When these variables are not controlled in the analysis, the effect of early intervention could be lost due to good outcomes in children, who despite having a late CI, had a pre-CI hearing experience [29]. Conversely, poor outcomes are possible in children with early implantation and diseases that limit their benefits [30, 31]. As in the present study, Conway et al. [13] only included children with bilateral profound hearing loss with no residual hearing prior to CI. They also found a significant negative correlation between implicit sequence learning abilities and age at implantation: children with the least auditory deprivation and earlier CI showed better visual sequence learning outcomes.

Furthermore, in the present study, a significant percentage (64%) of children were diagnosed before 6 months and received cochlear implant within 12 months of age (50%, between 8 and 10 months). All of these children reached normal performances (≥ 25° percentile) in attention flexibility and visuo-spatial working memory. Ninety-four percent of them also performed as expected for their chronological age in behavioral flexibility and inhibition control. On the other hand, children diagnosed and implanted later showed more variable performance, with 56% of them scoring < 25 percentile in inhibition control and in visuo-spatial working memory and < 25 percentile in both behavioral and attention flexibility. To our knowledge, this is a novel finding, as it underlines how receiving CI within 12 months of age influences EF skills when compared to children implanted later, in agreement with findings reported in the auditory and communicative domains [32]. One possible explanation is that earlier access to sound is fundamental to activating attention-demanding systems [13] and also to fully develop some basic cognitive mechanisms, important for learning, such as sensory integration [33]. In fact, the experience acquired in the early stages of life with sound and auditory patterns, which are complex signals and arranged in series, gives the child the opportunity to develop the bases for neurocognitive and executive functions such as: detection of patterns, sequential memory, sustained attention, cognitive flexibility, planning, and problem-solving [13, 34]. Early identification and early implantation significantly reduce deprivation and give children the opportunity to learn from exposure to complex auditory stimuli in the period of maximum brain plasticity [35].

Visuo-spatial working memory did not correlate either with age at diagnosis or with age at implantation. The children in the present study were all preschooler, with a maximum age of 6, and so were still using a visuo-spatial code—not affected by auditory deprivation—and this could explain the lack of correlation. Young children’s visuo-spatial working memory seems to rely above all on the ability of the child to visually store perceptive characteristics of the materials which they need to memorize [36]. They use information such as shape, orientation, and detailed appearance [36], but are not yet able to form a verbal mental model of the objects. This reliance on visual information is gradually replaced by the verbal rehearsal of visual and spatial cues of objects by age 7 [37].

Speech perception was also associated with all of the EF abilities studied, with the exception of the domain of inhibitory control. Beer et al. [10] did not investigate the relationship between EFs and speech perception in their sample of preschool children, while some connection was found in the few studies that focused on this aspect in school children. The relationship seems to be closely linked to the type of EF domain being investigated [8, 14, 18]: direct or inverse correlations were found with verbal working memory, verbal rehearsal speed [8, 14], and behavior regulation [18], but not with spatial working memory and inhibition concentration [8]. A possible explanation could be the differing complexities of the speech perception tasks being performed and the degree of verbal demand of each EF task. For example, speech in noise tasks is more complex than speech in quiet ones as the first is regulated by a child’s ability to focus attention on the speech signal, correctly encoding, storing, and reproducing words in the sentence while simultaneously inhibiting the distracting noise. Therefore, they are likely to be more effective in identifying relationships with various EF skills, such as inhibition–concentration [18].

Language skills correlated significantly with the EF domains, as already shown in the literature.

CI children with better language, as assessed through language tests, are more able to switch between verbal and perceptual domains, and between different sorting concepts within each of the domains [5, 16]. Furthermore, language helps children regulate behavior, concentrate, and inhibit impulsive responses [16]. Internal and external languages are used to reflect on events and tasks and it allows for the decoupling of action from reality. Direct behavior can, therefore, be guided by action plans, that are stored internally in the working memory, rather than by immediate external factors [18]. Poor language skills can, therefore, make it difficult to memorize the rules and the phases of a plan and can induce children to give more impulsive or hasty answers [15]. Unfortunately, the lack of an NH control group did not allow to investigate the direction of the relation.

The data in the literature on DHH children are, instead, conflicting about the relationship between visuo-spatial working memory and language. Spatial, visual, and language skills were positively correlated in the studies of Figueras et al. [16], Surowiecky [38], and Jones et al. [39]. In particular, a longitudinal study revealed a developmental path that suggests that visuo-spatial working memory does not develop optimally when the child’s existing vocabulary is weak [39]. In other studies, visuo-spatial WM was less dependent on the development of the first lexical organization and, therefore, more resistant to delays and early hearing disorders [7–9, 11]. Visuospatial WM tasks may differ in the degree of sequential processing of stimuli. Despite the seemingly non-verbal design of the task, children could still make use of verbal mnemonic strategies such as labelling stimuli or naming aloud the position of a stimulus [38]. The visuo-spatial task which we used in the present study may have benefited from these verbal strategies, and children with better language skills may have obtained higher scores through their use, thus helping to establish a positive correlation between language skills and visuo-spatial WM performance.

Gender seems to play a role only for the attention flexibility task: females appeared to have better skills in focusing attention on a mental set, resisting interference. Although there are no other studies on gender difference and EF performance in children with CI, studies focusing on children with NH have shown that girls would perform better in verbal working memory and attention, while boys would achieve better results in terms of spatial reasoning/working memory and cognitive flexibility [40].

The role of economic income has been poorly studied. In the present study group of preschool CI children, it was significantly correlated to flexibility and inhibitory abilities. This finding concurs with the outcomes described in school-aged DHH children by Mitchell and Quitner [41]. On the contrary, Beer et al. [10] found no correlation between the studied EFs and these socio-demographic variables. Studies on NH children have shown that low economic income is associated with worse performance in inhibitory control tasks, working memory, executive attention, as well as flexibility and planning [42]. The influence of economic income is already traceable in infants and pre-schoolers, and this persists over time [43]. Given the initial premises and results described in this work and in the one by Mitchell and Quitner [41], it would be important that future prospective studies investigate further the role of economic income in the FEs of children with CI.

Finally, in the present study, it was found that parental education level influences the EF skills of children with CI: children of parents with a higher education level have been positively associated with flexibility of response change and inhibitory control. This new finding correlates with the linguistic results described by Geers et al. [44] in a large population of CI children. Parental education level has been described as a very important factor influencing NH children's cognitive development, also predicting the correct development of EF performance [45]. Parents with a higher education level create a more stimulating environment from an intellectual point of view, establishing the environmental conditions that favor the development of EFs [45]. They talk more, read more often to their children, and use a richer vocabulary than parents with lower educational levels [46]. Children of parents with higher education levels tend to have a wider vocabulary, faster language development, and better performance in cognitive tests, including EFs [45].

Some constraints limit the generalization of current results. The sample was small and only limited statistical analysis could be performed. The EF assessment was performed solely through a direct test of the children and this limited the comparison with other studies that integrated parental behavior checklists to measure additional EF domains in daily life. Finally, the study assessed EF skills at a given time, preschool age, but longitudinal data are not yet available, leaving unanswered the question as to whether these skills are maintained at an older age, when basic competences mature and further and more complex skills will require a higher cognitive load.

## Conclusion

The results of this study highlight that early intervention, language development, economic income level, gender, and parental education all play a role in determining the development of EFs in preschool CI children. Most of the early implanted children developed good EF skills. However, some of them, particularly if implanted after 12 months of age, showed difficulties in all of the studied EFs.

Evaluation of EF skills should, therefore, become a routine aspect of follow-up immediately after cochlear implantation, and intervention programs should include strategies and specific training to enhance and monitor these skills. A timely identification of developmental difficulties, if present, could allow professionals to implement an appropriate stimulation program for the child, therefore indirectly helping families to put into practice behaviors able to enhance EF competences. Further longitudinal studies would be useful to add information on the variables that influence the development of EFs and to improve the effectiveness of intervention strategies.

## References

[1] Clark C, Prior M, Kinsella G (2002). The relationship between executive function abilities, adaptive behavior, and academic achievement in children with externalizing behavior problems. J Child Psychol Psychiatry.

[2] Carlson SM, Wang TS (2007). Inhibitory control and emotion regulation in preschool children. Cogn Dev.

[3] Petersen IT, Bates JE, Staples AD (2015). The role of language ability and self-regulation in the development of inattentive-hyperactive behavior problems. Dev Psychopathol.

[4] Baddeley A (2007) Working memory, thought, and action, Oxford psychology series, vol 45. Oxford University Press, London. 10.1093/acprof:oso/9780198528012.001.0001

[5] Remine MD, Care E, Brown PM (2008). Language ability and verbal and nonverbal executive functioning in deaf students communicating in spoken English. J Deaf Stud Deaf Educ.

[6] Conway CM, Pisoni DB, Kronenberger WG (2009). The importance of sound for cognitive sequencing abilities: the auditory scaffolding hypothesis. Curr Dir Psych Sc.

[7] Kronenberger WG, Pisoni DB, Henning SC, Colson BG (2013). Executive functioning skills in long-term users of cochlear implants: a case control study. J Pediatr Psychol.

[8] Kronenberger WG, Beer J, Castellanos I, Pisoni DB, Miyamoto RT (2014). Neurocognitive risk in children with cochlear implants. JAMA Otolaryngol Head Neck Surg.

[9] Kronenberger WG, Colson BG, Henning SC, Pisoni DB (2014). Executive functioning and speech-language skills following long-term use of cochlear implants. J Deaf Stud Deaf Educ.

[10] Beer J, Kronenberger WG, Castellanos I, Colson BG, Henning SC, Pisoni DB (2014). Executive functioning skills in preschool-age children with cochlear implants. J Speech Lang Hear Res.

[11] Castellanos I, Pisoni DB, Kronenberger WG, Beer J (2016). Early expressive language skills predict long-term neurocognitive outcomes in cochlear implant users: evidence from the MacArthur–Bates Communicative Development Inventories. Am J Speech Lang Pathol.

[12] Davidson LS, Geers AE, Hale S, Sommers MM, Brenner C, Spehar B (2019). Effects of early auditory deprivation on working memory and reasoning abilities in verbal and visuospatial domains for pediatric cochlear implant recipients. Ear Hear.

[13] Conway CM, Pisoni DB, Anaya EM, Karpicke J, Henning SC (2011). Implicit sequence learning in deaf children with cochlear implants. Dev Sci.

[14] Pisoni DB, Cleary M (2003). Measures of working memory span and verbal rehearsal speed in deaf children after cochlear implantation. Ear Hear.

[15] Botting N, Jones A, Marshall C, Denmark T, Atkinson J, Morgan G (2017). Nonverbal executive function is mediated by language: a study of deaf and hearing children. Child Dev.

[16] Figueras B, Edwards L, Langdon D (2008). Executive function and language in deaf children. J Deaf Stud Deaf Educ.

[17] Barker DH, Quittner AL, Fink NE, Eisenberg LS, Tobey EA, Niparko JK, CDaCI Investigative Team (2009). Predicting behavior problems in deaf and hearing children: the influences of language, attention, and parent-child communication. Dev Psychopathol.

[18] Beer J, Kronenberger WG, Pisoni DB (2011). Executive function in everyday life: implications for young cochlear implant users. Cochlear Implants Int.

[19] Hintermair M (2013). Executive functions and behavioral problems in deaf and hard-of-hearing students at general and special schools. J Deaf Stud Deaf Educ.

[20] Gioia GA, Espy KA, Isquith PK (2003). Behavior rating inventory of executive function-preschool version.

[21] Shoda Y, Mischel W, Peake PK (1990). Predicting adolescent cognitive and self-regulatory competencies from preschool delay of gratification: identifying diagnostic conditions. Dev Psychol.

[22] Kahlert J, Gribsholt SB, Gammelager H, Dekkers OM, Luta G (2017). Control of confounding in the analysis phase—an overview for clinicians. Clin Epidemiol.

[23] Raven J (2003) Raven progressive matrices. In: McCallum RS (ed) Handbook of nonverbal assessment. Springer, Boston, pp 223–237. 10.1007/978-1-4615-0153-4_11

[24] Cutugno F, Prosser S, Turrini M (2000). Audiometria Vocale.

[25] Gilmour L (2010) The inter-rater reliability of categories of auditory performance-II (CAP)-II. Masters Thesis. Institute of Sound and Vibration Research, University of Southampton

[26] Cianchetti C, Sannio Fancello G (1997). Test TVL. Test di valutazione del linguaggio. Livello prescolare.

[27] Rustioni D, Lancaster M (1994). Prove di comprensione della produzione linguistica—PVCL.

[28] Valeri G, Stievano P, Ferretti ML, Mariani E, Pieretti M (2015). Battery for assessment of executive functions-BAFE.

[29] Geers AE, Nicholas JG, Moog JS (2007). Estimating the influence of cochlear implantation on language development in children. Audiol Med.

[30] Demir B, Cesur S, Sahin A, Binnetoglu A, Ciprut A, Batman C (2019). Outcomes of cochlear implantation in children with inner ear malformations. Eur Arch Otorhinolaryngol.

[31] Shearer AE, Hansen MR (2019). Auditory synaptopathy, auditory neuropathy, and cochlear implantation. Laryngosc Investig Otolaryngol.

[32] Leigh J, Dettman S, Dowell R, Briggs R (2013). Communication development in children who receive a cochlear implant by 12 months of age. Otol Neurotol.

[33] Houston DM, Stewart J, Moberly A, Hollich G, Miyamoto RT (2012). Word learning in deaf children with cochlear implants: effects of early auditory experience. Dev Sci.

[34] Kral A, Kronenberger WG, Pisoni DB, O'Donoghue GM (2016). Neurocognitive factors in sensory restoration of early deafness: a connectome model. Lancet Neurol.

[35] Kuhl PK (2010). Brain mechanisms in early language acquisition. Neuron.

[36] Pickering SJ (2001). The development of visuo-spatial working memory. Memory.

[37] Fenner J, Heathcote D, Jerrams-Smith J (2000). The development of way finding competency: asymmetrical effects of visuo-spatial and verbal ability. J Environ Psychol.

[38] Surowiecki VN, Sarant J, Maruff P, Blamey PJ, Busby PA, Clark GM (2002). Cognitive processing in children using cochlear implants: the relationship between visual memory, attention, and executive functions and developing language skills. Ann Otol Rhinol Laryngol Suppl.

[39] Jones A, Atkinson J, Marshall C, Botting N, St Clair MC, Morgan G (2020). Expressive vocabulary predicts nonverbal executive function: a 2-year longitudinal study of deaf and hearing children. Child Dev.

[40] Lipina SJ, Martelli MI, Vuelta B, Colombo JA (2005) Performance on the A-not-B task of Argentinean infants from unsatisfied and satisfied basic needs homes. Interam. J Psychol 39:49–60 [fecha de Consulta 27 de Junio de 2020]. ISSN: 0034-9690. https://www.redalyc.org/articulo.oa?id=284/28439106

[41] Mitchell TV, Quittner AL (1996). Multimethod study of attention and behavior problems in hearing-impaired children. J Clin Child Psychol.

[42] Mezzacappa E (2004). Alerting, orienting, and executive attention: developmental properties and socio-demographic correlates in an epidemiological sample of young, urban children. Child Dev.

[43] Clearfield MW, Jedd KE (2013). The effects of socio-economic status on infant attention. Inf Child Dev.

[44] Geers AE, Nicholas JG, Sedey AL (2003). Language skills of children with early cochlear implantation. Ear Hear.

[45] Ardila A, Rosselli M, Matute E, Guajardo S (2005). The influence of the parents' educational level on the development of executive functions. Dev Neuropsychol.

[46] Hoff E (2003). The specificity of environmental influence: socio-economic status affects early vocabulary development via maternal speech. Child Dev.

